# Changes in the morphology and cell ultrastructure of a microalgal community exposed to a commercial glyphosate formulation and a toxigenic cyanobacterium

**DOI:** 10.3389/fmicb.2023.1195776

**Published:** 2023-06-22

**Authors:** Claudia Ivette Hernández-García, Fernando Martínez-Jerónimo

**Affiliations:** Instituto Politécnico Nacional, Escuela Nacional de Ciencias Biológicas, Lab. de Hidrobiología Experimental Carpio y Plan de Ayala S/N, Col. Santo Tomás, Mexico City, Mexico

**Keywords:** phytoplankton, herbicides, water pollution, harmful algal blooms, Faena^®^, cyanotoxins

## Abstract

Human activities significantly influence the health of aquatic ecosystems because many noxious chemical wastes are discharged into freshwater bodies. Intensive agriculture contributes to the deterioration by providing indirectly fertilizers, pesticides, and other agrochemicals that affect the aquatic biota. Glyphosate is one of the most used herbicides worldwide, and microalgae are particularly sensitive to its formulation, inducing displacement of some green microalgae from the phytoplankton that leads to alterations in the floristic composition, which fosters the abundance of cyanobacteria, some of which can be toxigenic. The combination of chemical stressors such as glyphosate and biological ones, like cyanotoxins and other secondary metabolites of cyanobacteria, could induce a combined effect potentially more noxious to microalgae, affecting not only their growth but also their physiology and morphology. In this study, we evaluated the combined effect of glyphosate (Faena^®^) and a toxigenic cyanobacterium on the morphology and ultrastructure of microalgae in an experimental phytoplankton community. For this purpose, *Microcystis aeruginosa* (a cosmopolitan cyanobacterium that forms harmful blooms) and the microalgae *Ankistrodesmus falcatus*, *Chlorella vulgaris*, *Pseudokirchneriella subcapitata*, and *Scenedesmus incrassatulus* were cultivated, individually and jointly, exposing them to sub-inhibitory concentrations of glyphosate (IC_10_, IC_20,_ and IC_40_). Effects were evaluated through scanning electron (SEM) and transmission electron (TEM) microscopy. Exposure to Faena^®^ produced alterations in the external morphology and ultrastructure of microalgae both individually and in combined cultures. SEM evidenced the loss of the typical shape and integrity of the cell wall and an increase in the biovolume. TEM revealed reduction and disorganization of the chloroplast, variation in starch and polyphosphate granules, formation of vesicles and vacuoles, cytoplasm degradation, and cell wall continuity loss. The presence of *M. aeruginosa* was, for microalgae, an additional stress factor adding to the chemical stress produced by Faena^®^, increasing the damage in their morphology and ultrastructure. These results alert to the effects that can be caused by glyphosate and the presence of toxigenic bacteria on the algal phytoplankton in contaminated and anthropic and eutrophic freshwater ecosystems.

## 1. Introduction

Human activities influence the health and conservation of aquatic ecosystems (CONAGUA, 2018), because they supply a large variety of pollutants that degrade their quality and affect the aquatic biota (Iummato et al., 2019), and lead to the loss of ecosystem services (Smith, 2003). Conservation of aquatic ecosystems is fundamental for the equilibrium and functionality of the trophic structure in these environments. Maintenance of primary production is essential because the phytoplankton provides energy and the necessary materials for higher trophic levels (Aguilar, 2003).

Intensification of agriculture induces the incorporation of agrochemicals and nutrients into freshwater bodies, causing contamination and eutrophication (Smith, 2003). The demand for food by the increasing human population has promoted the development of intensified agricultural practices associated with the need to increase the use of fertilizers and pesticides (CONAGUA, 2018), which, due to their mobilization in the environment, lixiviation or surface runoff, end up in freshwater bodies (Lozano et al., 2018; Iummato et al., 2019).

Eutrophication is characterized by an increase in the biomass of the phytoplankton due to the excessive availability of limiting nutrients in the water (O’Neil et al., 2012). The accelerated growth of the phytoplankton increases the biochemical demand of oxygen and hinders light penetration into more deep layers, causing anoxia (Smith, 2003). These conditions can predispose a change in the composition of the phytoplankton, favoring the loss of sensitive groups and increasing the presence of cyanobacteria (Cloern, 2001; Smith, 2003; Xu et al., 2010; Pérez-Morales et al., 2016) of genera like *Anabaena*, *Anabaenopsis*, *Cylindrospermopsis*, *Lyngbya*, *Microcystis*, *Nodularia*, *Phormidium*, *Planktothrix*, and *Pseudanabaena* (Komárek and Komárková-Legnerová, 2002; Quiroz-Castelan et al., 2004; Vasconcelos et al., 2010), many of them are potentially toxigenic.

The massive and accelerated growth of cyanobacteria is a phenomenon that occurs in many eutrophicated waterbodies in the world (Chorus and Welker, 2021), and when it is associated with the synthesis and release of cyanotoxins to the environment it is known as harmful cyanobacterial blooms (HCBs; Huisman et al., 2018; USEPA, 2023).

Among the most critical species forming HCBs is *Microcystis aeruginosa*, which strives successfully in eutrophication conditions (Huisman et al., 2018) and can produce microcystins. Cyanotoxins include a group of more than 90 alkaloids, peptides, and lipopolysaccharides (Van Apeldoorn et al., 2007; Puddick et al., 2013; Almanza, 2016).

Because cyanobacteria produce microcystins together with other bioactive secondary metabolites, they can damage the phytoplankton, especially green microalgae (Ishida and Murakami, 2000; Leão et al., 2009a). This damage can go from inhibiting their growth rates (Ishida and Murakami, 2000; Etchegaray et al., 2004; Suikkanen et al., 2004; Van Der Grinten et al., 2005; Beresovsky et al., 2006; Gantar et al., 2008; Dunker et al., 2013; Chia et al., 2018) to affecting their metabolism, inducing alterations of the antioxidant response (Pflugmacher et al., 2007), inhibiting photosynthesis (Beresovsky et al., 2006; Pflugmacher et al., 2007; Gantar et al., 2008), as well as morphological (Pflugmacher et al., 2007) and cell ultrastructure (Gantar et al., 2008; Martínez-Ruiz and Martínez-Jerónimo, 2018) alterations.

For this reason, HCBs are important from an ecological point of view because they can cause a change in the structure of the phytoplankton community and induce harmful effects on the aquatic biota. HCBs are also relevant from the public health perspective because of their implications in human health by the presence of cyanotoxins in the drinking water and, in the economic realm, due to the productive activities that can be affected (Hernández-García and Martínez-Jerónimo, 2020).

On the other side, several agrochemical products, in general, and herbicides, in particular, affect freshwater bodies (Relyea, 2005), either through a direct toxic effect or because of their bioaccumulation and circulation through the trophic levels (Lozano et al., 2018).

One of the most widely used herbicides worldwide is glyphosate [*N*-(phosphonomethyl) glycine] used to control weeds in agricultural, forestry, and urban environments (Borggaard and Gimsing, 2008). It is used in more than 160 countries (Woodburn, 2000) in chemical varieties that include salts of isopropylamine, ammonium, di-ammonium, dimethyl ammonium, and potassium (Benbrook, 2016).

After its application on the crop fields, glyphosate is decomposed in a short time (Borggaard and Gimsing, 2008). However, it has been found that under certain conditions, it can persist for prolonged periods (Bento et al., 2019) and be carried to waterbodies through surface run off and lixiviation (Lozano et al., 2018). To ease the input of glyphosate to the plant, the commercial formulations include surfactants (Tsui and Chu, 2003) such as polyoxyethylene amine (POEA) and MON 0818 (Van Bruggen et al., 2018), which are even more toxic than glyphosate (Vendrell et al., 2009; Wu et al., 2016).

Green microalgae, cyanobacteria, and some fungi share with plants the shikimate metabolic pathway, hence, glyphosate can affect them, diminishing their growth rates (Vendrell et al., 2009; Hernández-García and Martínez-Jerónimo, 2020), inducing oxidative stress (Álvarez-Góngora et al., 2012; Ostera et al., 2016; Hernández-García and Martínez-Jerónimo, 2020), metabolic alterations (Wu et al., 2016), and morphological and ultrastructural damage (Amorós et al., 2007; Qian et al., 2008).

Considering the ecological relevance of phytoplankton and the increasing use of glyphosate, relevant efforts have been made to determine the effect of this herbicide on waterbodies. However, these efforts have focused mainly on experiments with one toxicant at a time and almost always assessing the response of only one species, which leads to a partial understanding of the problem generated by chemical pollutants (Relyea, 2005).

Particularly for microalgae and cyanobacteria, several publications have described the impact of different formulations based on glyphosate (Pérez et al., 2007; Vera, 2011; Vera et al., 2012; Wu et al., 2016; Smedbol et al., 2018), reporting in all adverse toxic effects. Likewise, the impact of anthropogenic eutrophication and possible participation of glyphosate in the development of HCBs has been investigated, as well as the change in the ecological dynamics of the phytoplankton due to them (Leão et al., 2009b; Berry et al., 2011; Felpeto and Vasconcelos, 2016; Pérez-Morales et al., 2016; Chia et al., 2018). However, few studies approach the problem from an integrated perspective that considers the effect of several chemical stressors on a phytoplankton community. Even more scarce are the studies approaching the impact on the morphology and ultrastructure of phytoplankton as a means to understand the damage caused by xenobiotics.

Because glyphosate is well-identified as a chemical stressor that modifies the growth of microalgae and cyanobacteria and produces oxidative stress, it may also induce ultrastructural changes in microalgae. On the other hand, cyanobacteria are a dominant group of eutrophic freshwater ecosystems; their capacity to synthesize toxic bioactive metabolites is also known, hence they could also produce stress in microalgae, affecting their ultrastructure and altering their growth and physiology.

Considering the latter, the combined effect of chemical stressors of different nature and origin could produce changes at the cellular level, which would imply changes in the population dynamics of the phytoplankton.

Therefore, this work analyzed the changes in the external morphology and the ultrastructure of an experimental community of Chlorophycean microalgae (*Ankistrodesmus falcatus*, *Chlorella vulgaris*, *Pseudokirchneriella subcapitata*, and *Scenedesmus incrassatulus*), in the presence of the herbicide glyphosate (Faena®) and of a toxigenic variety of the *Microcystis aeruginosa* cyanobacterium as an additional stress factor. The rationale is that microalgae can be affected by biological stressors, such as toxigenic cyanobacterium, that could produce morphological and ultrastructural damages, increasing the toxicity of chemical stressors, such as the herbicide Faena®. These changes were evaluated through scanning and transmission electron microscopy (SEM and TEM).

## 2. Materials and methods

### 2.1. Culture conditions

Microalgae and the cyanobacterium were cultivated in Bold’s Basal Medium (250 g of NaNO_3_, 25 g of CaCl₂ 2H₂O, 75 g of MgSO₄∙7H₂O, 7.35 g of K_2_HPO_4,_ 175 g of KHPO_4_, 25 g of NaCl, 1 ml of H_2_SO_4_, 4.98 g of FeSO_4_∙7H_2_O, 11.42 g of H_3_BO_3_ in 1,000 ml of deionized water, without adding the micronutrients solution nor EDTA; Bold, 1942)_._ Strains were obtained from the Microalgae and Cyanobacteria Collection of the Experimental Hydrobiology Laboratory of the *Escuela Nacional de Ciencias Biológicas, Instituto Politécnico Nacional*. The test species were the toxigenic cyanobacterium *Microcystis aeruginosa* (VU5 strain) and the Chlorophycean *Pseudokirchneriella subcapitata*, *Ankistrodesmus falcatus*, *Chlorella vulgaris*, and *Scenedesmus incrassatulus*. All these species had remarkable differences in size and shape, avoiding confusion during their identification in mixed cultures. In each case, the initial cultures were made with a 0.2 mg L^−1^ inoculum, incubated at 25°C under continuous light conditions (85 μmoles photons m^−2^ s^−1^), and constant aeration.

### 2.2. Subinhibitory toxicity assays

To evaluate the toxicity of glyphosate (Faena®), the inhibitory concentration (IC) values equivalent to IC_40_, IC_20_, and IC_10_, reported in Hernández-García and Martínez-Jerónimo (2020) for the commercial Faena® formulation were used (Table 1). These concentrations were below the median growth inhibition. They were chosen to determine if these amounts of the active ingredient and adjuvants contained in Faena® produce changes in the ultrastructure and identify the morphological and ultrastructural alterations at low concentrations. Bioassays were started with 0.2 mg L^−1^ inoculum in individual assays and the experimental community. Differences in the shape and size of the cells allowed easily identifying the species and discrimination of the changes in morphology and internal ultrastructure. Exposures were made in 250-ml Erlenmeyer flasks with 100 ml of the test solution, using Bold’s Basal as a dilution medium.

**Table 1:** Inhibitory concentrations (96 h) reported by Hernández-García and Martínez-Jerónimo (2020) for microalgae and *M. aeruginosa* exposed to the herbicide Faena^®^.

	*A. falcatus*	*P. subcapitata*	*C. vulgaris*	*S. incrassatulus*	*M. aeruginosa*
	mg L^−1^	mg L^−1^	mg L^−1^	mg L^−1^	mg L^−1^
IC_10_	0.488	0.203	0.696	0.397	0.204
IC_20_	0.703	0.353	0.984	0.767	0.404
IC_40_	1.144	0.743	1.564	1.851	1.005
IC_50_	1.411	1.022	1.908	2.702	1.486

Values are for the equivalent to glyphosate concentrations in the commercial formulation.

The effects were measured for each microalga, and the cyanobacterium through individual culture assays under the described Faena® concentrations (IC_10_, IC_20_, and IC_40_). After this, the microalgal experimental community was exposed to four Faena® concentrations equivalent to the IC_40_, determined for each microalgal species, with and without *M. aeruginosa*. All assays were performed for 96 h incubating at 25°C, with continuous illumination of 85 μmols photons m^−2^ s^−1^, without aeration.

### 2.3. Scanning electron microscopy observations

In all cases and in both individual and mixed cultures, after 96 h of incubation, the cultures were centrifuged at 3,000 rpm for 15 min. The cell pellet was washed in deionized water and resuspended in a phosphate buffer solution (pH 7.4). Cells were fixed with 2.5% glutaraldehyde for 2 h and then washed three times for 5 min with phosphate buffer solution (pH 7.4). Then, 2% osmium tetroxide was applied for 1 h for an additional fixing of cells, which were washed three times with phosphate buffer solution for 5 min each time. Cell pellets were dehydrated following an ethanol gradient (30, 40, 50, 60, 70, 80, and 90%) for 10 min in each solution, ending with three immersions in absolute ethanol for 10 min. A critical point dryer was used, and the samples were covered with gold powder. Observations were made at a 15 kV acceleration voltage.

### 2.4. Transmission electron microscopy observations

The SEM sample preparation method was followed until the complete dehydration with absolute ethanol. Then, propylene oxide was used two times during 20 min each time. Samples were embedded in resin through a propylene oxide-resin gradient (3:1, 1:1, 2:1) for 24 h each; finally, they were embedded in 100% resin for 2 h and making an exchange for polymerizing the resin at 62°C during 48 h. Cuts were made with an ultramicrotome with a thickness of 70 nm and were contrasted with uranyl acetate and lead citrate; observations were made with a 60-kV acceleration voltage.

## 3. Results

### 3.1. SEM observations

#### 3.1.1. Individual cultures

**Figure 1:**
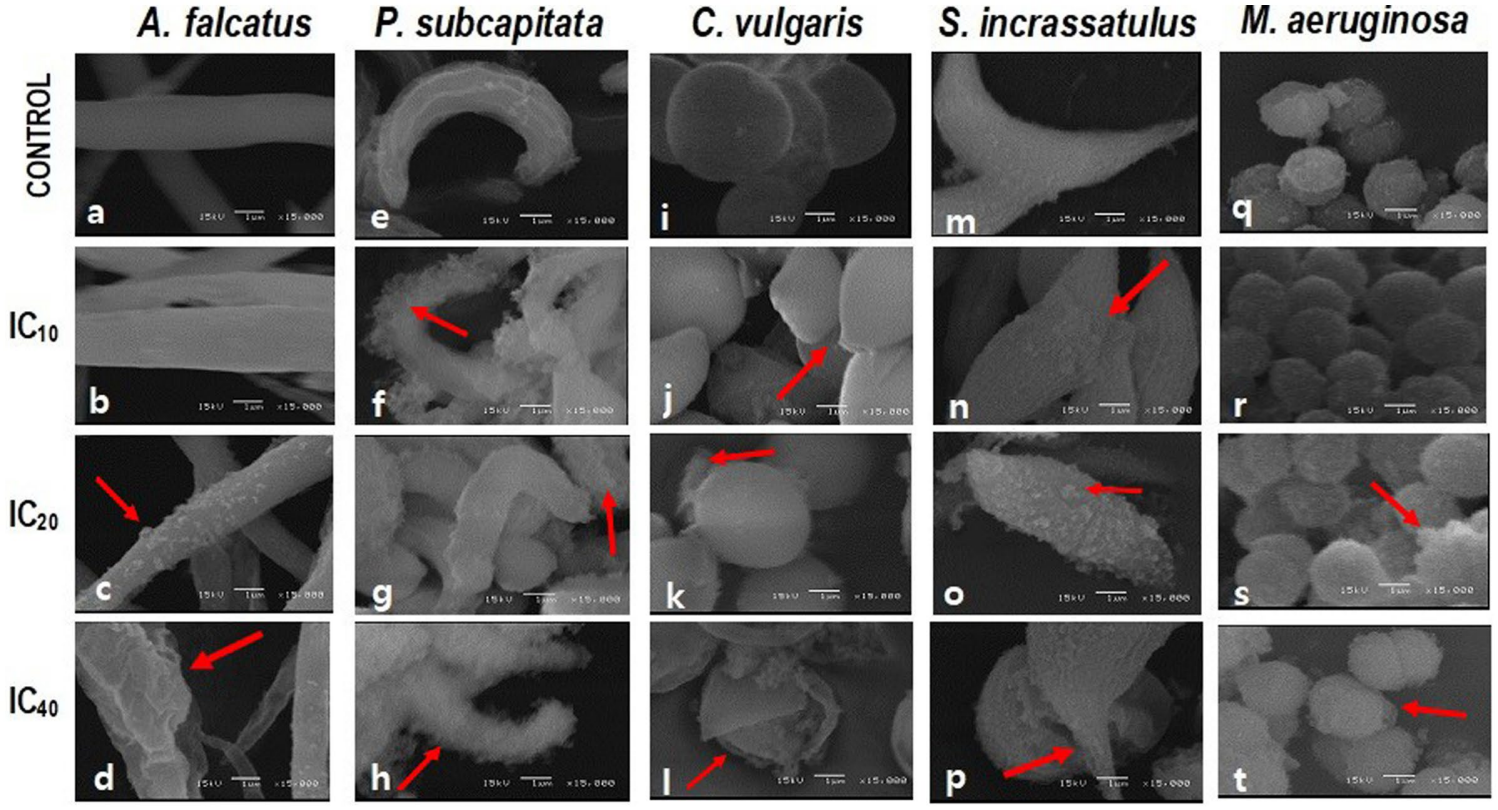
SEM microphotographs showing the changes in cell morphology of *M. aeruginosa* (*M.a*) and microalgae in individual cultures after exposure to glyphosate (Faena^®^) during 96 h at sub-inhibitory concentrations (IC_10_, IC_20_, and IC_40_). **(a–d)** *A. falcatus* (*A.f*), desquamation of the cell wall; **(e–h)** *P. subcapitata* (*P.s*), loss of cell wall integrity when increasing the herbicide concentration; **(i–l)** *C. vulgaris* (*C.v*), rupture of the cell wall and loss of the characteristic spheric shape; and **(m–p)** *S. incrassatulus* (*S.i*), desquamation of cells and appearance of rifts on the cell surface. **(q–t)** *M. aeruginosa* alteration of the cell wall.

In all cases, the cell damage increased in magnitude and frequency when increasing the Faena® concentration (Figure 1). Under normal conditions, *A. falcatus* showed a spiculate shape with a smooth cell wall (Figure 1A). Whereas with the IC_10_, the cell wall showed rifting (Figure 1B), and with the IC_20_, desquamation was observed (Figure 1C) with deformation of the cellular ends. With the IC_40_, the cell size increased, and the shape was altered, presenting frequent cells with folded ends; the discontinuous cell wall presented squamous adherences (Figure 1D).

The *P. subcapitata* reference cells showed a half-moon shape with longitudinal rifts (Figure 1E). Exposure to Faena® produced a fibrous material of spongy aspect around the cell (Figures 1F–H).

Control *C. vulgaris* showed a spherical shape and smooth cell wall (Figure 1I), whereas, with the IC_10_, cells became ovoid with a slightly pointed end (Figure 1J). Starting with the IC_20_, the spongy fibrous material around the cell was observed (Figures 1K,L). With the IC_40_, rupture, cell wall detachment, and cell membrane disaggregation were observed (Figure 1L).

Typically, *S. incrassatulus* control cells clustered in coenobia of four cells of roughly the same size; after exposure to Faena®, a gradual loss of the aggregation capacity that led to individual cells was observed. With the IC_10_, the cell wall presented desquamation and rifting (Figure 1N). Deterioration of the cell wall with an increase in the biovolume was observed from the IC_20_ (Figures 1O,P) on, whereas with the IC_40_, cells with thinning of one end and balloon-type in the other were observed (Figure 1P).

*Microcystis aeruginosa*, like the microalgae, showed a gradual deterioration of the cell wall proportionally to the herbicide’s concentration; the typical spherical shape was substituted by the ovoid shape (Figures 1R,T).

#### 3.1.2. Joint culture of microalgae

**Figure 2:**
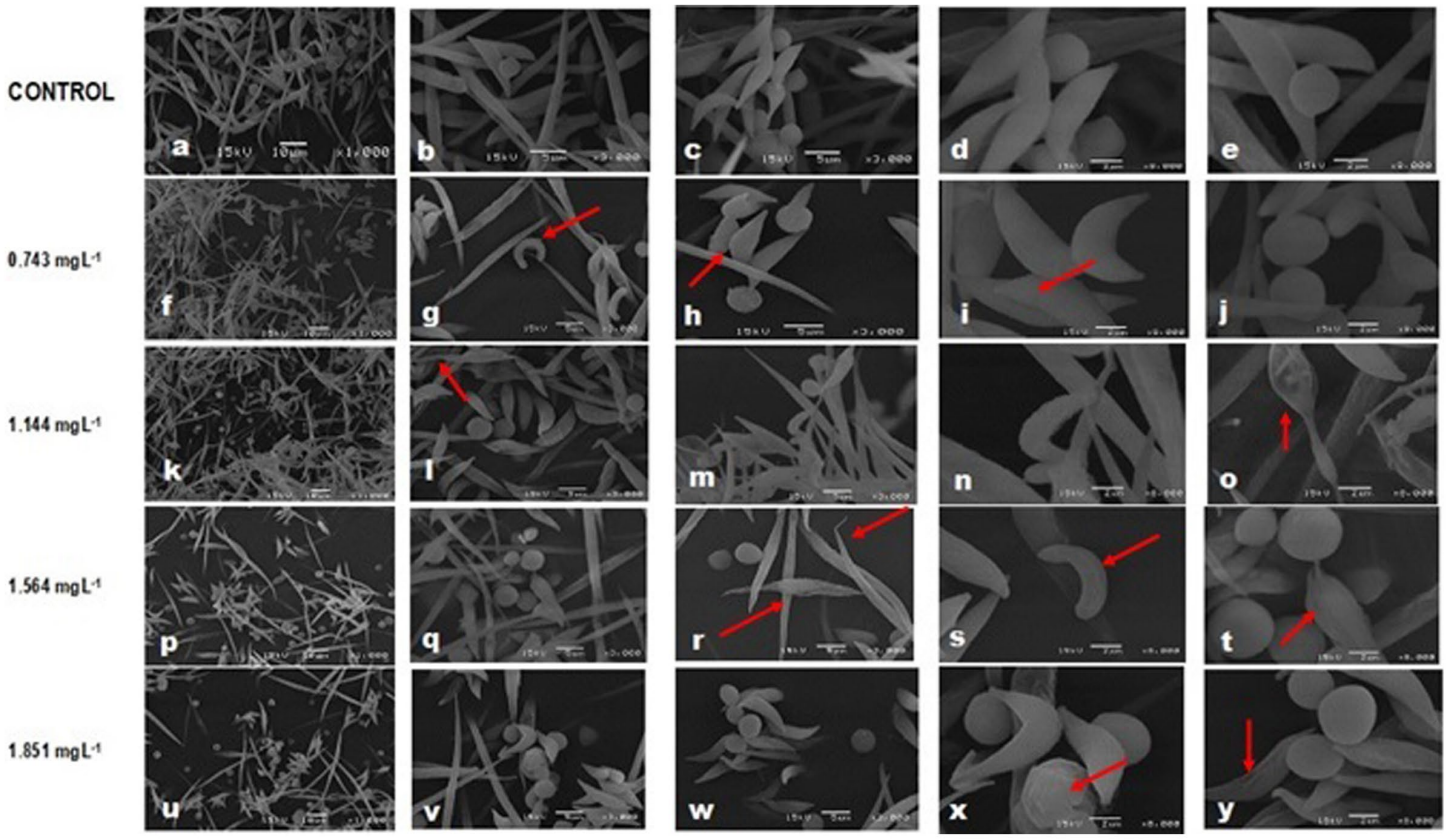
Changes in cell morphology of microalgae in combined cultures after exposure to glyphosate (Faena^®^) during 96 h. **(a–e)** Control culture. **(f–j)** *A.f*, folded tips and cell wall desquamation; *P.s*, irregular surface of the cell wall; *S.i*, individual cells with rifts; and *C.v*, ovoid cells; **(k–o)** *A.f*, thinning of one end; *P.s*, damaged cell wall; *S.i*, deep rifts; and *C.v*, ovoid form; **(p–t)** *A.f*, thinning in the mid-region. Similar damage in the rest of the species to those reported for the former concentration. **(u–y)** *A.f,* deformities in all cells; *P.s* and *S.i*, rifts and *C.v*, detachment of the cell wall.

Most cells of the controls were normal, with a morphology coinciding with that described for the individual control bioassay, although in about 10% of the small morphological changes were observed in some species: *A. falcatus* had desquamations and slightly curved ends, *C. vulgaris* depicted an ovoid shape with one slightly pointed end, whereas rifts were observed in *P. subcapitata* (Figures 2A–E). No alterations were observed in *S. incrassatulus*.

In the IC_40_ equivalent concentration for *P. subcapitata* (0.743 mg L^−1^), *A. falcatus* depicted cells with a thinned end, folded tips, and a cell wall with desquamation, whereas *P. subcapitata* showed an irregular cell wall. In *S. incrassatulus,* individual cells thinned in one of their ends predominately, with rifts in their longitudinal axis. *C. vulgaris* maintained its smooth cell wall without apparent damage, although its shape changed to an ovoid with a pointed end (Figures 2F–J).

At the 1.144 mg L^−1^ concentration (Figures 2K–O) corresponding to the IC_40_ of *A. falcatus*, *P. subcapitata* presented severe damage in its cell wall compared to *C. vulgaris,* which depicted scarce damage only changing to the ovoid change. The *S. incrassatulus* cells depicted deeper and more abundant rifts than with the former concentration. *A. falcatus* lost the spiculate shape and presented one thinned end, and the other was thicker; its cell wall presented squamous detachment.

Damage to the cell wall was evident in all microalgae starting at 1.564 mg L^−1^ (equivalent to the IC_40_ of *C. vulgaris*). Notably, *A. falcatus* showed significant changes, as it presented thinning in its mid-region, forming an asymmetric eight with thinned ends (Figures 2P–T).

At 1.864 mg L^−1^, equivalent to the IC_40_ of *S. incrassatulus*, a significant deterioration of the cell wall was observed in all cells, clearly greater than that described for the previously described concentrations. Additionally, *C. vulgaris* showed cell wall detachment and ovoid cells with a pointed end. *A. falcatus* depicted substantial damage and cell wall deformation, *P. subcapitata* and *S. incrassatulus* presented large rifts along their longitudinal axis (Figures 2U–Y). With this concentration, no normal cells were recorded.

#### 3.1.3. Combined culture of the algal community and *Microcystis aeruginosa*

**Figure 3:**
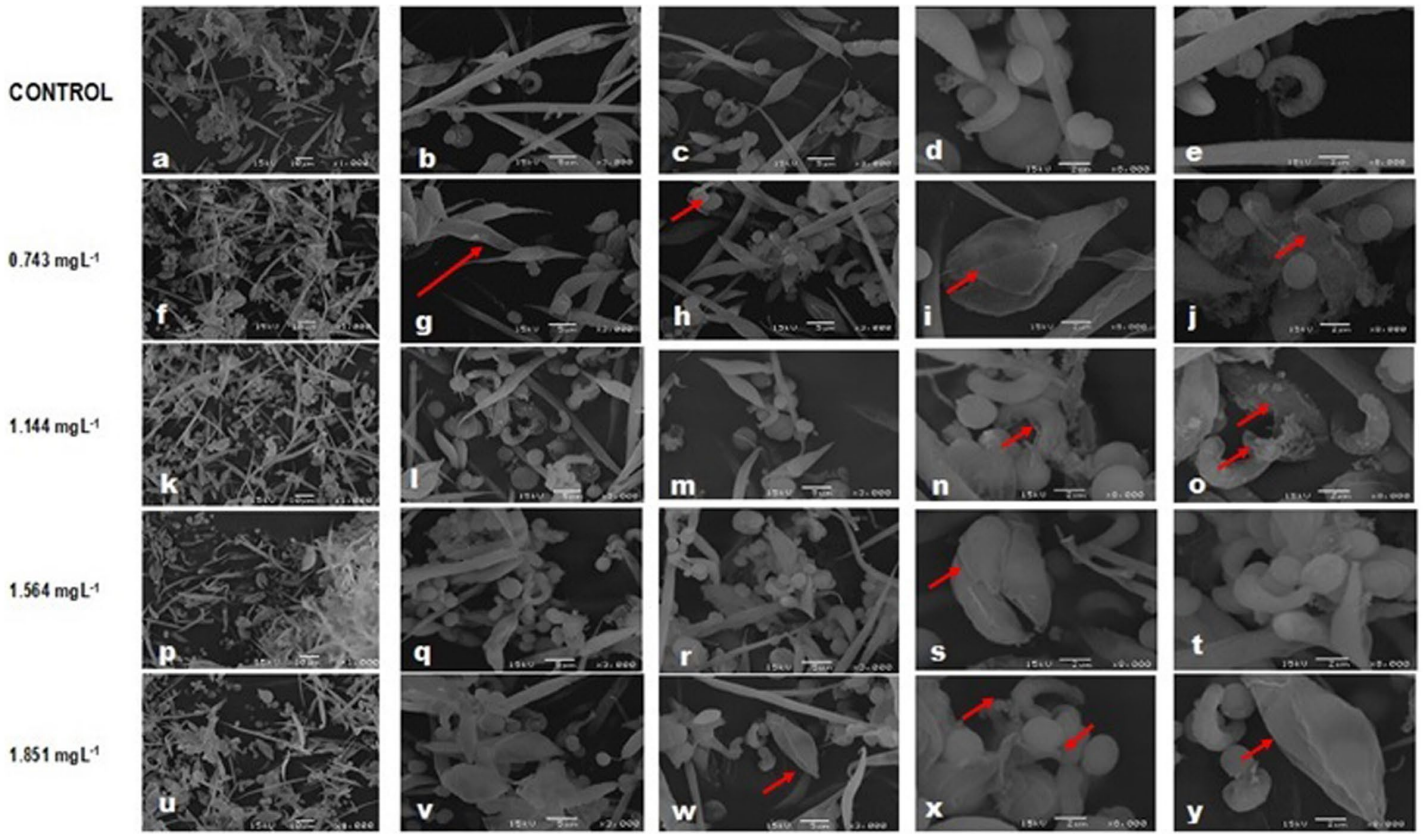
SEM images showing affectations in the cell morphology of a community of microalgae and cyanobacteria after exposure to glyphosate (Faena^®^) during 96 h at sub-inhibitory concentrations, IC_40_ of each species. **(a–e)** all microalgae species showed morphological alterations probably due to the actions of cyanotoxins. **(f–j)** Damage is mainly evident in the cell wall. **(p–y)** Desquamation of the cell wall, loss of the typical shape, and change in the biovolume.

All the microalgal species in the control bioassay (Figures 3A–E) showed morphological alteration despite not being exposed to Faena®. *A. falcatus* showed thinning in its end, narrowing in the mid-region, and a squamous cell wall. *P. subcapitata* presented rifts, with some cells of fibrous-spongy superficial aspect. *C. vulgaris* was observed with a rupture of the cell wall and an elliptic shape. *S. incrassatulus* did not present the development of coenobia.

At the 0.743 mg L^−1^ concentration, more damage was observed than those described at the same concentration when microalgae were grown alone (Figures 3F–J).

Desquamation was the main damage observed in the cell wall with increases in the toxicant exposure (Figures 3P–Y). The observed deformations are consistent with those already described; in *A. falcatus*, tips were thin and curved, and cells showed narrowing of the mid-region, forming a sort of asymmetric eight. In *C. vulgaris,* the cell wall became detached (Figures 3P–Y), whereas in *S. incrassatulus* the volume increased, with severe desquamation of the cell wall. The morphological changes of *M. aeruginosa* were gradual, adopting an ovoid shape. At the higher concentrations, peripheral clusters of amorphous material of fibrous-spongy aspect were formed.

### 3.2. TEM observations

#### 3.2.1. Individual cultures

For more clarity, results are presented per species.

##### 3.2.1.1. Ankistrodesmus falcatus

**Figure 4:**
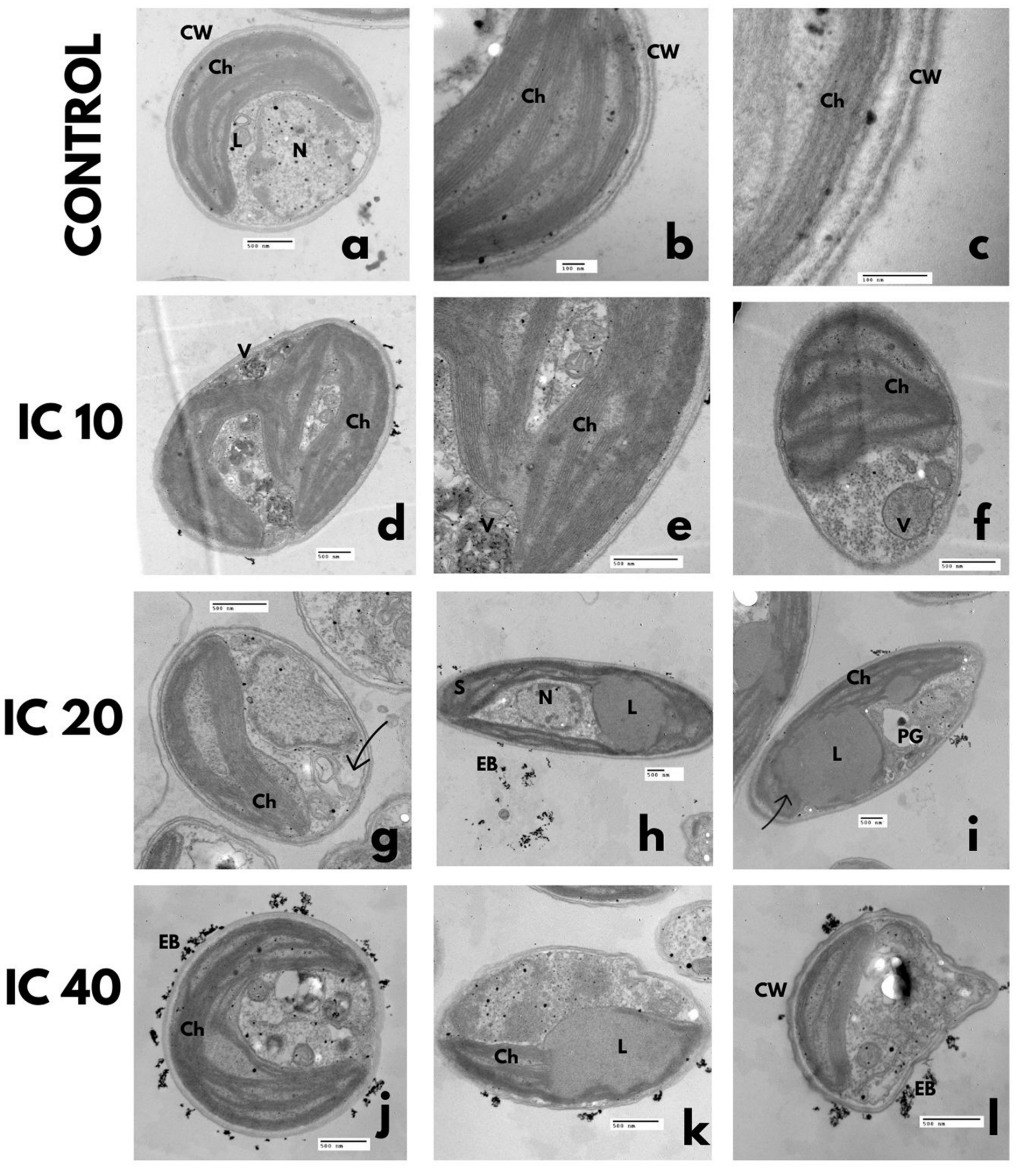
Effect on the ultrastructure of *A. falcatus* after exposure to Faena^®^ during 96 h at sub-inhibitory concentrations (IC_10_, IC_20_, and IC_40_). **(a–c)** Control, **(d–f)** disorganized chloroplast, electrodense bodies, degraded cytoplasm enveloped in double-membrane vacuoles, **(g–i)** disaggregated cytoplasm, increase of double-membrane vacuoles with electrodense bodies inside of them, reduced chloroplast with starch granules inside, **(j–l)** undulated cell wall, lipid vesicles of large size, deposition of electrodense bodies outside the cell. Observed structures; chloroplasts (Ch), vacuoles (V), electrodense bodies (EB), starch granules (S), nucleus (N), polyphosphate granules (PG), and lipid vesicles (L).

In the control, the cytoplasm was uniform and granulated, with a large parietal chloroplast, scarce polyphosphate and starch granules, and lipid vesicles. The nuclear membrane was well defined, with one nucleolus and condensed chromatin in the walls (Figures 4A,B). The cell was trilaminar uniform and thick (Figure 4C).

The obtained microphotographs after exposure to the IC_10_ shown in Figures 4D–F evidence double-membrane vesicles with electrodense bodies and a disorganized chloroplast, with starch granules in the stroma. The cytoplasm was observed to be slightly degraded, enveloped in double-membrane vacuoles, with a significant increase in the number and size of lipid vesicles, accompanied by a reduction in the number of polyphosphate granules.

Cells exposed to the IC_20_ presented a reduced and disorganized chloroplast with starch granules in its interior (Figures 4G–I). The increase in the number of phosphate granules and the degradation of the cytoplasm was evident, which led to the development of multiple double-membrane vesicles with electrodense bodies.

With the IC_40_, cells with an undulated wall were observed (Figure 4L), which developed large-size lipid vesicles that occupied up to half the cell volume; polyphosphate and starch granules increased but were of reduced size. The chloroplast was disorganized and reduced, and the cytoplasm was remarkably degraded. Deposition of electrodense bodies outside the cell occurred (Figure 4J).

##### 3.2.1.2. Pseudokirchneriella subcapitata

**Figure 5:**
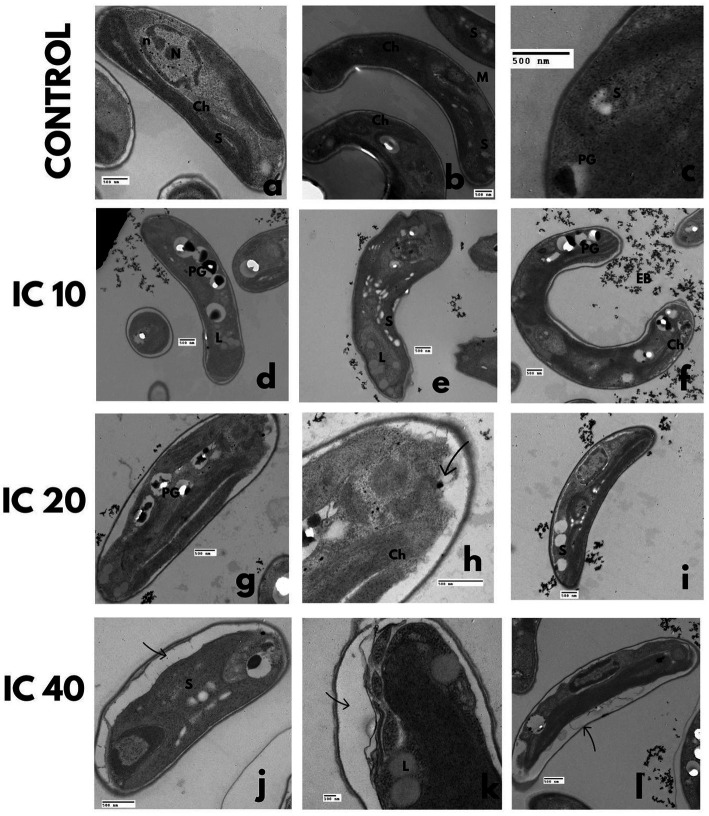
TEM microphotographs. Effect on the *P. subcapitata* ultrastructure after exposure during 96 h to Faena^®^ at sub-inhibitory concentrations. **(a–c)** Control, **(d–f)** IC_10_, increase of starch and polyphosphate granules, **(g–i)** IC_20_, degraded cytoplasm, **(j–l)** IC_40_, lipid vesicles, degraded cytoplasm. Observed structures: chloroplasts (Ch), vacuoles (V), electrodense bodies (EB), starch granules (S), nucleus (N), nucleolus (n), polyphosphate granules (PG), lipid vesicles (L), and cell wall (CW).

In the control bioassay, the nucleus and nucleolus were well-defined, with condensed chromatin in the walls of the nuclear membrane (Figure 5A). The cytoplasm was dense with mitochondria, starch, and polyphosphate granules (Figure 5C), and the chloroplast occupied two-thirds of the cellular volume (Figure 5B).

The increase in the toxicant concentration increased the starch and polyphosphate granules (Figures 5D–L), as well as the formation of lipid vesicles (Figure 5K). The exposure to the toxicant also produced degradation of the cytoplasm, loss of cell membrane continuity, and wall folding (Figures 5D–L). A reduction of the chloroplast and partial disorganization of the thylakoids (Figures 5J–L) occurred, as well as the formation of double-membrane vesicles that enveloped part of the cytoplasm with electrodense bodies in their interior. Accumulation of electrodense bodies in the exterior of cells was more conspicuous with the IC_10_ (Figures 5D–F).

##### 3.2.1.3. Chlorella vulgaris

**Figure 6:**
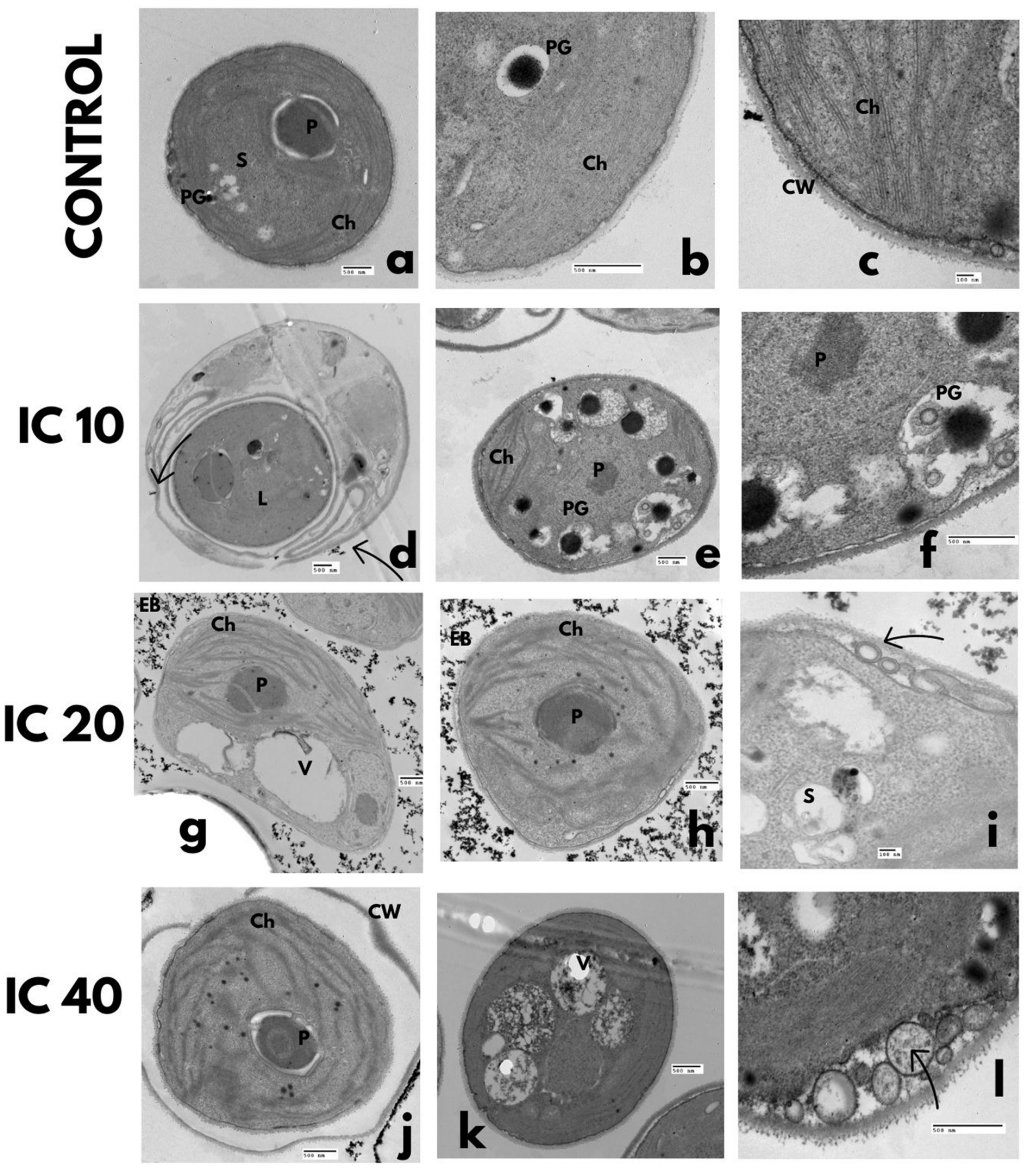
TEM microphotographs. *C. vulgaris* after Faena^®^ exposure during 96 h at sub-inhibitory concentrations (IC_10_, IC_20_, and IC_40_). **(a–c)** Control, **(d–f)** lipid vesicles and polyphosphate granules, **(g–i)** disorganized chloroplasts and vesicles between the membrane and the cell wall, **(J–L)** rupture of the cell wall and deposition of electrodense bodies inside the cell. Observed structures: chloroplasts (Ch), pyrenoids (P), vacuoles (V), electrodense bodies (EB), starch granules (S), nucleus (N), nucleolus (n), polyphosphate granules (PG), lipid vesicles (L), and cell wall (CW).

Control cells presented a thick and smooth wall (Figures 6A–C), a dense cytoplasm without electrodense materials, a large size chloroplast, a central pyrenoid with scarce starch granules, and thylakoids arranged along the central axis.

The progressive increase in Faena® concentration caused undulation of the cell membrane, degradation of the cytoplasm (Figures 6D–L), formation of lipid vesicles (Figure 6D), and an increase in polyphosphate granules. At the IC_20_ and IC_40_, vesicles were observed between the membrane and the cell wall, as well as an increase in the size and number of vacuoles.

Chloroplasts and pyrenoids were reduced, and the thylakoids became disorganized (Figures 6D–L). Rupture of the cell membrane was observed with leaks of the cytosol, aside from the deposition of electrodense material outside the cell and aggregation in groups of four cells (Figures 6G–L).

##### 3.2.1.4. Scenedesmus incrassatulus

**Figure 7:**
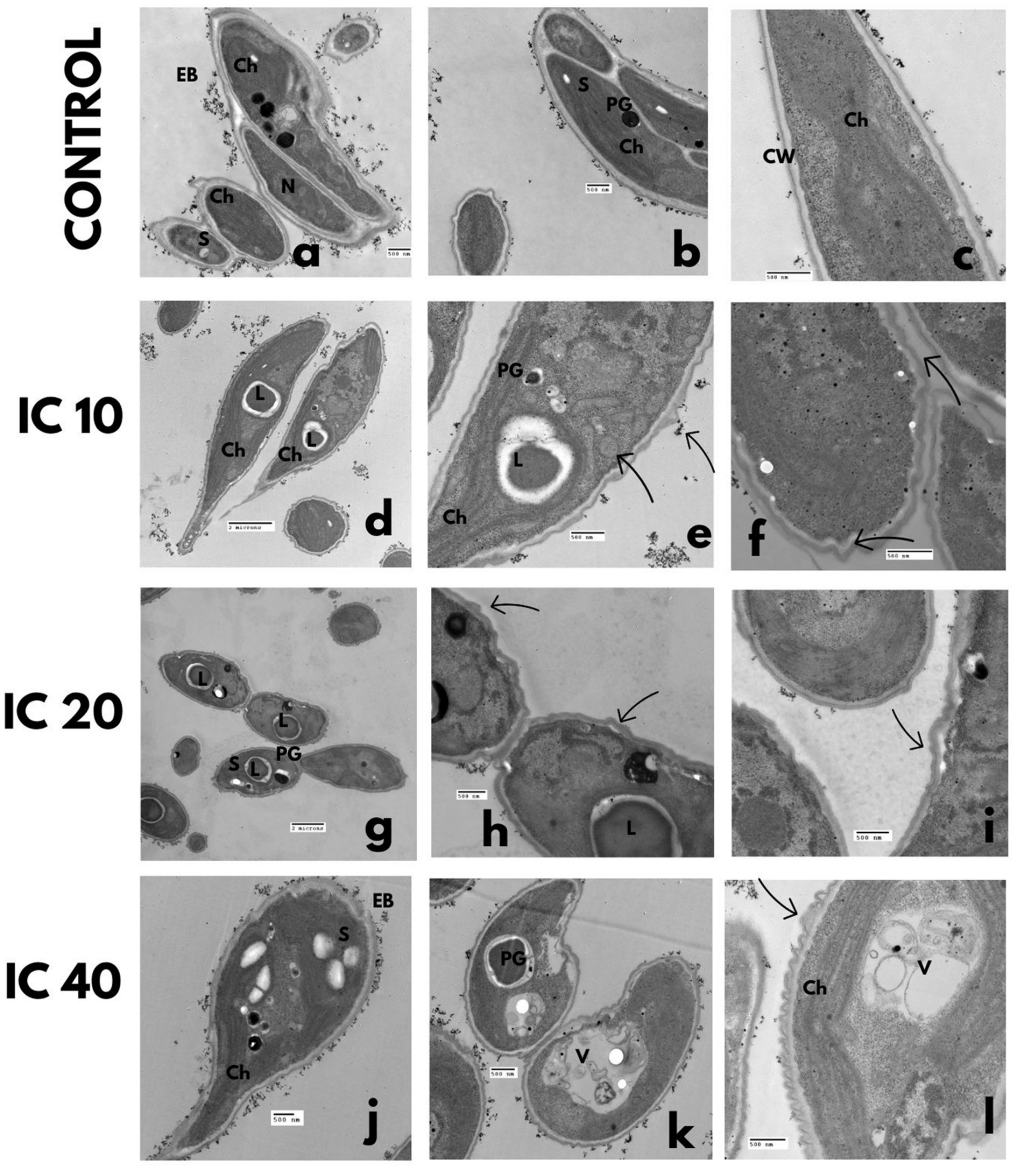
Ultrastructural changes of *S. incrassatulus.* TEM microphotographs after exposure to Faena^®^ during 96 h at sub-inhibitory concentrations (IC_10_, IC_20_, and IC_40_). **(a–c)** Control, **(d–f)** lipid vesicles and folding of the cell wall, **(g–i)** loss of cell wall continuity and lipid vesicles, **(j–l)** damage to the chloroplast, and loss of the typical shape. Observed structures: chloroplasts (Ch), vacuoles (V), electrodense bodies (EB), starch granules (S), nucleus (N), polyphosphate granules (PG), lipid vesicles (L), and cell wall (CW).

In the absence of the herbicide, cells were organized in coenobia of four same-sized cells, with continuous membrane and cell wall without folding, a dense cytoplasm with scarce starch and polyphosphate granules (Figures 7A–C), a parietal chloroplast and one pyrenoid.

The gradual increase in herbicide’s concentration induced loss of coenobium grouping, increase in cell volume, and the number of lipid vesicles and vacuoles (Figures 7D–L). Thinning of one end and a balloon-type shape in the opposite end of each cell were observed (Figure 7J). The cell wall became deformed, observing increasingly more conspicuous undulations (Figure 7L). With the IC_40_, the chloroplast, and the pyrenoid became reduced in size (Figures 7J–L).

##### 3.2.1.5. Microcystis aeruginosa

**Figure 8:**
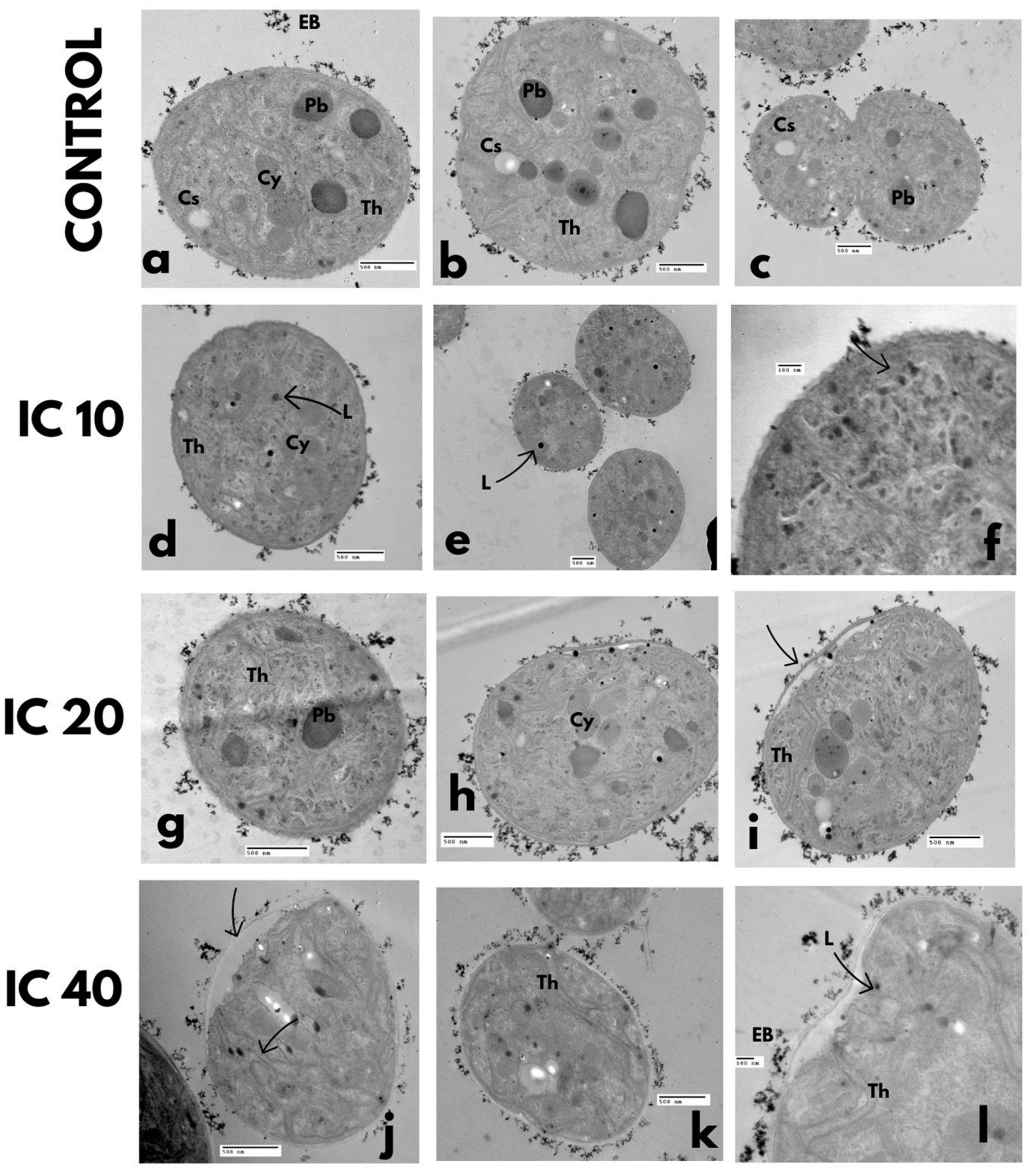
Effect on the ultrastructure of *M. aeruginosa* after exposure to Faena^®^ during 96 h at sub-inhibitory concentrations (IC_10_, IC_20_, and IC_40_). TEM microphotographs. **(a–c)** Control, **(d–f)** increase of lipid granules, **(g–i)** electrodense bodies in the periphery of cells, **(j–l)** thickening and loss of cell wall continuity. Observed structures: electrodense bodies (EB), polyhedral bodies (Pb), cyanophycin (Cy), cyanophycean starch (Cs), thylakoids (Th), and lipids (L).

Cells not exposed to Faena® presented a regular cell wall, dense cytoplasm, cyanophycin granules, cyanophycean starch, and polyhedral bodies (Figures 8A–C), parallel thylakoids distributed in the whole cytosol (Figure 8B), with electrodense bodies surrounding the cell wall (Figure 8A).

In the cells exposed to the IC_10_ concentration, a diminution in polyhedral bodies and an increase in tiny lipid granules in the cytosol were observed (Figures 8D–E). At the IC_20_, the electrodense bodies accumulated in the periphery of the cell wall increased (Figure 8G), and deterioration of thylakoids occurred (Figure 8I). The changes observed with the IC_40_ were related to a deformation and thickening of the cell wall (Figures 8J–L), as well as with the generalized degradation of the cytoplasm and the loss of the spheric shape (Figures 8J,L); the polyhedral bodies and the cyanophycin vesicles were scarce in the cells exposed to the Faena® concentration.

## 4. Discussion

Morphological and ultrastructural changes of microalgae and cyanobacteria exposed to Faena® increased in magnitude and frequency with increasing herbicide concentrations. In the individual bioassays with the species, microalgae and cyanobacteria underwent less damage than that observed in mixed algal cultures and the community of microalgae + cyanobacteria, observing in the latter the most extensive morphological and ultrastructural changes.

Loss of cell wall integrity, the increase in biovolume (in *A. falcatus* and *S. incrassatulus*), and the loss of the typical shape (in *C. vulgaris*, *S. incrassatulus*, and *M. aeruginosa*) provoked by Faena® were the main changes observed in the individual cultures with SEM.

Changes recorded in the cell wall could be explained as a consequence of reactive oxygen species (ROS) production by the glyphosate effect or by other ingredients in the Faena® formulation; consequently, the generated oxidative stress could have induced an imbalance in the enzymatic defense response, allowing ROS to reach and degrade the macromolecules that constitute the cell wall (Amorós et al., 2007; Álvarez-Góngora et al., 2012; Ostera et al., 2016; Hernández-García and Martínez-Jerónimo, 2020).

Xenobiotics and other chemical stressors can produce changes in the cell wall of microalgae and cyanobacteria. Iummato et al. (2019) reported thickening of the cell wall in *Scenedesmus vacuolatus* when exposed to Atanor® (glyphosate formulation); likewise, Asselborn et al. (2015) found damage to the cell wall when exposing *S. capricornutum* (*P. subcapitata*) to the chlorpyrifos insecticide.

In this study, the documented damage to the cell wall included deformations, desquamation, rifts, disintegration, and rupture. These alterations can be attributed to the toxic effects produced by exposure to Faena®.

In this regard, Lipok et al. (2010), using the Roundup® (glyphosate formulation) and the active principle, reported the IC_50_ for eight microalgal and cyanobacterial species, attaining growth inhibition at very variable intervals, from 2.9 to 598.4 mg L^−1^, pointing out that the commercial formulations were more toxic than the active ingredient. In that same study, authors reported that microalgae were more sensitive than cyanobacteria. Comparison of the present study with that of Lipok et al. (2010), who reported that Roundup® produced greater growth inhibition in *C. vulgaris* and *M. aeruginosa* because the IC_50_ determined by them was 118.1 and 6.7 mg L^−1^, respectively, whereas in our it was 1.908 and 1.486 mg L^−1^. These results evidence that sensitivity depends on the species, although the different formulations of the toxicant also exert an influence.

Another factor to be considered is the cell wall composition because, despite that all the microalgae studied herein were chlorophycean, there are bilaminar species and others that have a trilaminar structure with algaenan within this group, which makes them less permeable (Zych et al., 2009); hence, the damage could be related with the capacity of the cell wall to act as a limiting barrier in the passive transport of toxicants (Pereira et al., 2013).

The observed increase in cell volume is consistent with the increase in the biovolume reported in other microalgae exposed to xenobiotics and organophosphorus insecticides. Kent and Weinberger (1991) reported an increase of biovolume in *S. capricornutum, Chlamydomonas segnis*, and *Chlorella pyrenoidosa* when exposed to phenyl lithium. Likewise, Walsh and Alexander (1980) and Asselborn et al. (2000) described, respectively, the increase in cell volume in the diatom *Skeletonema costatum* exposed to ethoprop and chlorpyrifos; the latter could be explained as a consequence of the cellular division inhibition that could lead to the accumulation of macromolecules inside the cell (Kent and Weinberger, 1991). Hernández-García and Martínez-Jerónimo (2020) described an increase in the concentration of macromolecules in chlorophycean microalgae exposed to glyphosate (Faena®), which could be correlated to the increased volume found in this study.

Changes in the volume of other eukaryote cells exposed to toxicants have also been described; Lal and Saxena (1980) reported this effect after exposure to organochlorinated compounds. De Chacin (1984) observed similar changes due to carbamates in *Chlamydomonas segnis*. Finally, Asselborn et al. (2015) confirmed the increase in the size of *A. gracilis* cells exposed to chlorpyrifos.

Aside from the physiological and metabolic implications of the biovolume variation caused by the chemical stressors, the change in cell size could impact the foraging processes of the filtering zooplankton. This could alter the trophic networks affecting predators, changing the community compositions and the possible accumulation of phytoplanktonic biomass, with negative ecological consequences (Asselborn et al., 2015).

Exposure to xenobiotics affects the phytoplankton ecologically and physiologically because other parameters dependent on cell size, like the photosynthetic rate, absorption of nutrients, and the speed at which the water column deepens (Asselborn et al., 2015) can also be affected.

Martínez-Ruiz and Martínez-Jerónimo (2015) described the depolarization of the membrane due to the oxidative stress produced by nickel in *A. falcatus*; hence, it could be possible that glyphosate and cyanotoxins, by promoting oxidative stress, influence the concentration of macromolecules, altering the carbohydrates that act as intermediaries in diverse photosynthetic and respiratory pathways, leading to the formation of membrane and cell wall constituents. Their diminution could influence the loss of the typical shape of cells and contribute to the depolarization of the cell membrane. In this regard, Gómez et al. (2015) observed in Cu-exposed *Ulva compresa* a diminution in the performance of the photosystem II, which produces calcium entry to the cell, inducing depolarization of the membrane. It is possible that the ingredients of Faena® and the cyanotoxins exerted the same effect because they are also inhibitors of photosystem II in microalgae (Hernández-García and Martínez-Jerónimo, 2020).

The TEM microphotographs showed, in all studied species, a reduction of the size of chloroplasts accompanied by their disorganization and progressive degradation according to the increase in the toxicant concentration. Noticeable was also the increase of starch granules within the stroma at the intermediate concentration (IC_10_ and IC_20_) and their almost elimination at the IC_40_ in the degraded chloroplasts; in *C. vulgaris* and *S. incrassatulus*, the pyrenoid was also reduced.

Other studies have reported damage in the chloroplast after exposure to xenobiotics. Iummato et al. (2019) reported an accumulation of starch granules in the stroma and disorganization of the chloroplast in *S. vacuolatus* exposed to Atanor® (glyphosate); Carfagna et al. (2013) observed deformations, disordered thylakoids, reduced pyrenoid, and the presence of starch granules in *Chlorella sorokiniana* exposed to cadmium and lead.

Carotenes are part of the cell protection systems that helps to eliminate ROS. Oxidate stress can affect carotenogenesis (Okamoto et al., 2001; Salguero et al., 2003), reducing the content of this photosynthetic accessory pigment. The reduction in carotenes could result in lesser oxygen (singlet and free) deactivation efficiency and an inability to halt the lipoperoxidation chain reactions provoked by ROS. Therefore, the degradation of chloroplasts and the reduction of the pyrenoid observed herein could be related to the changes in the concentration of the photosynthetic pigment that limits the antioxidant defense of the cell.

Hernández-García and Martínez-Jerónimo (2020) confirmed that microalgae and cyanobacteria exposed to glyphosate (Faena®) diminish their production of pigments associated with oxidative stress, suggesting that a possible effect of *M. aeruginosa* cyanotoxins on the microalgae diminishes the photosynthetic pigments concentration and increases cellular lipoperoxidation due to oxidative stress.

Destabilization of the compounds between proteins and pigments in thylakoidal membranes could be another explanation for the chloroplast degradation (Telfer et al., 1996; Domonkos et al., 2013), tied to the low concentration of chlorophylls (Hernández-García and Martínez-Jerónimo, 2020) and the imbalance of the antioxidant system. Lee and Hsu (2014) demonstrated changes in the thylakoid piling pattern due to the oxidative damage induced by high temperatures and correlated this event with the oxidative damage caused by other chemical stressors like herbicides, which agrees with the results presented herein.

Lin et al. (2013) stated that one of the primary sources of ROS could be the chloroplasts because, under stress conditions, the flow of electrons becomes irregular in the transporting chain, which induces oxidative stress and could particularly influence the disorganization of thylakoids. Results reported by De María et al. (2005) and Liu and Xiong (2009) on the ultrastructure of chloroplasts and thylakoids in microalgae are similar to those found in this study.

Degradation of the cytoplasm and formation of double-membrane vesicles observed with TEM could be associated with processes similar to autophagy, which is considered a sort of programmed cell death that can occur due to stress in microalgae (Affenzeller et al., 2009). Evidence of autophagy exists after the formation of double-membrane vesicles as reported by Crespo et al. (2005) in *Chlamydomonas reinhardtii* exposed to rapamycin (macrocyclic lactone that acts as an immuno-suppressor) and by Sicko-Goad et al. (1989) in *Cyclotella meneghiniana* exposed to chlorinated benzenes. Conversely, Affenzeller et al. (2009) attributed the appearance of double-membrane vesicles in *Micrasterias denticulata* to saline stress. In our study, it is possible that these vesicles were formed once the cell and cytoplasm organelles were degraded, causing the endoplasmic reticulum to envelope the degraded material, which could be related to the biovolume increase. Other studies have described this event in several programmed cell death studies in response to diverse chemical stressors (Kroemer et al., 2005; Van Doorn and Woltering, 2005).

The excretion and isolation mechanisms used by microalgae to compartmentalize the toxicants could explain the formation of vacuoles, which store the biotransformation products to attenuate the damage to macromolecules (Qian et al., 2008). These vacuoles could also contain residues of the degraded cytoplasm and organelles during lipoperoxidation. The development of vacuoles in microalgae subjected to different types of stress could also be related to programmed cell death. Affenzeller et al. (2009) and Ning et al. (2002) observed a marked vacuolization in *M. denticulata* and *Anabaena* sp. under saline stress conditions. Darehshouri et al. (2008) described the presence of vacuoles in *Micrasterias* sp. that had been exposed to H_2_O_2_. Asselborn et al. (2015) reported the presence of vacuoles in *A. gracilis* exposed to chlorpyrifos, and Iummato et al. (2019) described vacuoles in *S. vacuolatus* after exposing them to glyphosate in the commercial Atanor® formulation. This phenomenon has also been observed in our study in several microalgae exposed to sub-inhibitory concentrations of glyphosate (Faena®).

The increase in starch granules could be caused by interruption of the respiratory chain in mitochondria due to oxidative stress and possibly also by the exposure to the cyanotoxins released by *M. aeruginosa*, triggering the accumulation in chloroplasts and the loss of thylakoids organization by alterations of mitochondrial activity (Peixoto, 2005).

The appearance and increase of starch in microalgae under stress conditions were reported by Lebsky (2004). Visviki and Rachlin (1994), Nishikawa et al. (2003), and Volland et al. (2011) also observed that microalgae formed starch granules in the presence of metals. Wong and Chang (1988) and Asselborn et al. (2015) observed similar changes when exposing microalgae to organophosphorus insecticides.

The detoxification mechanisms (Volland et al., 2011; Carfagna et al., 2013) and those related to energy reserves accumulation (Yu et al., 2011) are known responses to stress used by microalgae to limit the damage (Carfagna et al., 2013). Visviki and Rachlin (1994), Nishikawa et al. (2003), Volland et al. (2011), and Zhang et al. (2020) reported that microalgae in the presence of metals formed lipid vesicles. Likewise, Wong and Chang (1988) and Asselborn et al. (2015) observed the formulation of lipid clusters after exposing microalgae to organophosphorus insecticides. Tukaj et al. (1998) confirmed the presence of lipid bodies in *Scenedesmus* in the presence of diesel oil.

Hernández-García and Martínez-Jerónimo (2020) reported an increase in the concentration of lipids in the same microalgal species used in this study, demonstrating that the oxidative stress caused by Faena® can be a triggering factor of the increase in lipids, which was confirmed through TEM.

Saçan et al. (2007) mention that the variation in the number of polyphosphate granules of different microalgae exposed to chemical stressors depends on the species and the toxicant’s concentration. Our results agree with the aforementioned because polyphosphate granules increased with IC_10_ and IC_20_, although they diminished with IC_40_ compared to the control cell. The increase of these granules can be interpreted as a defense mechanism of the cells; however, at high concentrations (IC_40_), this protection mechanism is overridden, hence, their presence diminished, perhaps due to generalized damage in cell metabolism.

It is probable that the starch granules, aside from being an energy reserve, act together with polyphosphates as detoxifiers (Asselborn et al., 2015) through the hydrolysis of phosphates, which, by forming short orthophosphate chains, allow the binding of their functional groups to the toxicants inactivating them (Nishikawa et al., 2003).

*M. aeruginosa* depicted a diminution of polyhedral bodies, possibly caused by the oxidative stress promoted by the herbicide affecting photosynthesis. The polyhedral bodies store the ribulose bisphosphate carboxylase (RuBisCO) enzyme, probably affecting the metabolic route of photosynthesis, specifically in the Calvin cycle, where RuBisCO has a fundamental role in CO_2_ fixation.

As for microalgae, the development of electrodense bodies inside and outside the cell was observed in *M. aeruginosa*. These particles could be polyphosphates, acting as detoxifiers by using their active site to immobilize the herbicide (Nishikawa et al., 2003).

The morphological and internal cell structure effects due to exposure to toxic compounds, like Cu, glyphosate, and the additives included in the herbicide’s formulation, affect the cellular physiology that could be revealed as negative functional changes with consequences at the population level and in the dynamics of the community. Likewise, the *in-situ* development of other chemical stressors, like cyanotoxins and other secondary metabolites produced by cyanobacteria like *M. aeruginosa*, alert on the sum of consequences of the chemical pollutants on the environmental deterioration observed in the freshwater bodies anthropically eutrophicated. Documentation of the morphological and ultrastructural alterations, which were species-specific, allows suggesting damage in the phytoplankton community that would lead to the potential deterioration and affectation of the phytoplankton-feeding species and the whole trophic structure in those environments.

## 5. Conclusion

Exposure to the commercial glyphosate formulation (Faena®) produced toxic effects predominantly in microalgae, although the cyanobacterium was also affected, despite it not being a target species. The synthesis and release of cyanotoxins by *M. aeruginosa* contributed to increasing the stress in microalgae. Both stressors modified the structure of the experimental community due to the structural and intracellular damage shown differentially by the exposed species.

The main effects observed with SEM were loss of the cell wall integrity, loss of the typical shape, and an increase of the biovolume, whereas TEM revealed changes in the size of the chloroplast and the pyrenoid, as well as degradation and disorganization of thylakoids. Changes in the number of starch and polyphosphate vacuoles, vesicles, and granules were also evident. These changes were found in individual and combined cultures. However, under combined culture (microalgae + cyanobacteria), microalgae were affected both by the herbicide and the possible production of microcystins and other secondary metabolites with biological activity because the damage was significantly more significant in this combined culture.

The damage observed in this study alerts us about the current worldwide use of glyphosate because of the impact that could be produced on the aquatic biota and, particularly, on the primary producers in anthropically eutrophicated environments. As shown, sub-inhibitory concentrations can influence not only the population growth of microalgae but also induce damage to their ultrastructure and metabolism. These observations ratify the need to legislate the use of glyphosate because the impacts on non-target organisms, like those of the phytoplankton, are highly relevant, impacting the trophic structure of aquatic environments.

The presence of chemical pollutants in aquatic ecosystems can contribute to the predominance of cyanobacteria and the potential development of HCBs, degrading, even more, the water quality and representing an additional risk for the use of the water resource.

## Data availability statement

The original contributions presented in the study are included in the article/supplementary material, further inquiries can be directed to the corresponding author.

## Author contributions

FMJ developed the concept of the study. CIHG and FMJ were responsible for the tests, data collection, and analysis. CIHG wrote the initial draft. Both authors approved the manuscript for publication.

## Conflict of interest

The authors declare that the research was conducted in the absence of any commercial or financial relationships that could be construed as a potential conflict of interest.

## Publisher’s note

All claims expressed in this article are solely those of the authors and do not necessarily represent those of their affiliated organizations, or those of the publisher, the editors and the reviewers. Any product that may be evaluated in this article, or claim that may be made by its manufacturer, is not guaranteed or endorsed by the publisher.
